# lmerSeq: an R package for analyzing transformed RNA-Seq data with linear mixed effects models

**DOI:** 10.1186/s12859-022-05019-9

**Published:** 2022-11-16

**Authors:** Brian E. Vestal, Elizabeth Wynn, Camille M. Moore

**Affiliations:** 1 Center for Genes, Environment and Health, National Jewish Health, 1400 Jackson St, Denver, CO 80206 USA; 2 Department of Biostatistics and Informatics, University of Colorado Denver, Anschutz Medical Campus, Aurora, CO USA

**Keywords:** RNA-Seq, Linear mixed models, Correlated data

## Abstract

**Background:**

Studies that utilize RNA Sequencing (RNA-Seq) in conjunction with designs that introduce dependence between observations (e.g. longitudinal sampling) require specialized analysis tools to accommodate this additional complexity. This R package contains a set of utilities to fit linear mixed effects models to transformed RNA-Seq counts that properly account for this dependence when performing statistical analyses.

**Results:**

In a simulation study comparing lmerSeq and two existing methodologies that also work with transformed RNA-Seq counts, we found that lmerSeq was comprehensively better in terms of nominal error rate control and statistical power.

**Conclusions:**

Existing R packages for analyzing transformed RNA-Seq data with linear mixed models are limited in the variance structures they allow and/or the transformation methods they support. The lmerSeq package offers more flexibility in both of these areas and gave substantially better results in our simulations.

## Background

Increasingly, RNA sequencing (RNA-Seq) studies utilize complex designs that induce correlation between observations. Examples include repeatedly measuring subjects over time, sampling related family members, or examining multiple tissue types from the same subject [1–4]. As the models used in the most popular R packages, edgeR [5] and DESeq2 [6], are not appropriate in these scenarios [7], several new approaches have been proposed. In general, these methods either (1) model the RNA-Seq counts directly using generalized linear mixed models (GLMMs) [8–10], or (2) transform the counts into continuous measures that can then be analyzed using linear mixed models (LMMs) assuming a normal distribution [11, 12]. The former approach has the benefit of modeling the RNA-Seq data directly, but model convergence and type 1 error rate control can be problematic at the smaller samples sizes common in RNA-Seq studies, depending on the GLMM estimation approach [8, 13]. The alternative of using transformed counts is appealing since LMMs have been extensively studied and are generally faster to fit. However, there are some drawbacks to this strategy as theoretical results show that it is not possible to stabilize variance via *any* transformation when the counts are too small [14], and thus the prospect that a mean-variance relationship persists in the transformed data can violate modeling assumptions in the downstream analyses. Moreover, the additional step of transforming the digital counts into a continuous measure has the potential to obscure relationships present in the original count data (e.g. higher power for MCMSeq compared to using linear mixed models on transformed data in [8] and more accurate estimates of heritability using GLMMs than with transformed data in [15]).

In Vestal et al. [8], we proposed the MCMSeq methodology to model correlated RNA-Seq data using a Bayesian hierarchical GLMM. Of the methods compared, MCMSeq best maintained nominal false discovery rates (FDRs) while providing strong sensitivity or statistical power. In addition, LMMs fit to variance stabilizing transformed (VST) data were the only other approach that controlled FDRs at their nominal level. While the sensitivity of this alternative was lower than MCMSeq, LMMs are substantially faster to fit and offer increased modeling flexibility compared to the current version of the mcmseq R package through the use of multiple random effects and correlation structures for residual errors. Consequently, we have developed the lmerSeq R package to fit gene-specific LMMs to transformed RNA-Seq data and easily generate results tables for either single regression coefficients or linear combinations of regression coefficients (contrasts). As an example that illustrates how the choices of transformation, model structure, and testing procedure can drastically alter inference, we compare the results from the VST-lmerSeq pipeline to two similar methods that rely on the VOOM transform: DREAM from the VariancePartion package and rmRNAseq from the eponymous package [11, 12, 16].

## Implementation

### Model

Like other transformation based approaches, lmerSeq uses a linear mixed model framework to analyze normalized counts. Let ${\varvec{Y}}_{gi} = \{Y_{gi1},\ldots , Y_{gin_{i}}\}$ be a vector of transformed expression values for gene *g* from subject *i* at observations 1 to $n_{i}$. Transformed expression is modeled using a LMM framework, so that ${\varvec{Y}}_{gi} = {\varvec{X}}_{i}{\varvec{\beta }}_{{\varvec{g}}} + {\varvec{Z}}_{i}{\varvec{b}}_{{\varvec{gi}}} + {\varvec{\epsilon }}_{gi}$. Here, ${\varvec{\beta }}_{{\varvec{g}}}$ is a $p \times 1$ vector of fixed effect regression coefficients for gene *g*, ${\varvec{X}}_{i}$ is a $n_{i} \times p$ matrix of fixed effects covariates for subject *i*, ${\varvec{b}}_{gi}$ is a $q \times 1$ vector of random effects for gene *g* and subject *i*, ${\varvec{Z}}_{i}$ is a $n_{i} \times q$ matrix of random effect covariates for subject *i*, and ${\varvec{\epsilon }}_{gi}$ is a a $n_{i} \times 1$ vector of normally distributed residual error terms with mean 0 and covariance $\Sigma _{g}$.

This model can account for correlation between samples in two ways, through the use of random effects (i.e. using ${\varvec{Z}}_{i}$ and ${\varvec{b}}_{gi}$) or through the covariance structure for the error terms, $\Sigma _g$. DREAM utilizes random effects, while rmRNAseq models the residual correlation directly by assuming a continuous autoregressive framework (CAR) for $\Sigma _{g}$ without the inclusion of random effects. Both of rmRNAseq and DREAM use the VOOM transformation, which estimates a mean-variance relationship for log-transformed counts in conjunction with a precision weight for each observation. lmerSeq allows either random effects or modeling of $\Sigma _g$ directly using a variety of covariance structures. While the lmerSeq functions are general enough to handle any data transformation (and weights), in the results presented below we utilized the VST available from the DESeq2 package to remove the relationship between the mean and variance, producing data that are approximately log_2_ scaled [6].

### lmerSeq R package

The lmerSeq package is written entirely in R and interfaces with the lme4, lmerTest, and nlme packages to fit the gene-specific LMMs [17–19]. lmerSeq users can fit models including multiple random effects, implement several of the correlation structures available in the nlme R package, perform a variety of tests, including constructing contrasts and simultaneous tests of multiple regression coefficients, and utilize multiple methods for calculating denominator degrees of freedom for F- and t-tests. Specifically, there are two model fitting functions, one that interfaces with lmerTest R package to fit linear mixed models with random effects and another that interfaces with the nlme R package to fit models with non-independent error covariance structures. In the former, any combination of random effects that are supported by lme4 can be used (e.g. random intercept, random slope, nested and/or crossed random effects, etc.), while in the latter support for compound symmetric and unstructured residual covariance structures is available. Parallel computing is supported on Mac and Linux operating systems via forking through the mclapply function from the Parallel R package. For both versions of the model fitting functions we have implemented summary functions that return results in a similar format to other RNA-Seq analysis packages like edgeR and DESeq2. These summaries can be made for individual regression coefficients, simple linear contrasts (i.e. one dimensional contrasts with t-tests), or simultaneous tests of multiple regression coefficients or linear contrasts with F-tests. The lmerTest package offers both Satterthwaite and Kenward-Rogers methods for calculating degrees of freedom for test statistics, both of which are supported in lmerSeq models with random effects. We have also implemented the Satterthwaite method for use with the two correlation structures supported from the nlme package. In combination, the fit and summary functions also allow the users to run various model diagnostics. The summary function identifies genes that had singular fits (e.g. the random intercept variance was estimated to be 0), and an option allows the user to exclude these genes from the results tables. The list returned by the fitting function stores the complete fit objects for each individual gene, and this allows the user access to a myriad of diagnostic options available from various other R packages, including testing the residuals for heteroskedasticity and/or normality. Finally, the package contains a detailed vignette with several examples using simulated data.

### Simulation study

To compare error rate control and sensitivity, we conducted a comprehensive simulation study considering 2 basic study designs: (1) a 2 group (e.g. treatment and control) design with paired observations (baseline and follow up) for each subject; and (2) a 2 group design with repeated measurements at 4 time points for each subject. In both cases, we simulated RNA-Seq counts from a negative binomial GLMM as follows:

$$\begin{aligned} C_{gij}\sim & {} {\mathcal{N}\mathcal{B}}(\mu _{gij}, \alpha _{g}) \\ \log (\mu _{gij})= & {} \beta _{g0} + \beta _{g1} I_{T_{i}} + \beta _{g2}t_{ij} + \beta _{g3}I_{T_{i}} t_{ij} + {\varvec{Z}}_{ij}{\varvec{b}}_{gi} \\ {\varvec{b}}_{gi}\sim & {} {\mathcal {N}}(0, \Sigma _{gb}) \end{aligned}$$

where $C_{gij}$ is the observed counts of gene *g* for subject *i* at observation *j*, $I_{T_{i}}$ is a treatment group indicator for subject *i* that equals 1 if the subject is in the treatment group and 0 else, $t_{ij}$ is the observation time (0 for baseline and 1 for the single follow up in simulation scenario 1 and 1–3 for the 3 follow up time points in scenario 2). For each of the sample sizes evaluated (N = 3, 5, 10, or 20 per group), 10 datasets with approximately 15k genes each were simulated using triplets of baseline expression ($\beta _{g0}$), random intercept variance ($\Sigma _{gb}$), and dispersion ($\alpha _{g}$) estimated from multiple human RNA-Seq datasets with repeated measures [2, 3, 20].

In Simulation Scenario 1, a random intercept was used to create correlation between the repeated measures, while Scenario 2 utilized both a random intercept and slope, with the standard deviation of the random slope set to 30% of the random intercept standard deviation, resulting in a more complex correlation structure. $\beta _{g1}$ and $\beta _{g2}$ were set to 0. In each dataset, we simulated differential expression for 20% of the genes by setting $\beta _{g3} \ne 0$ as follows: for Scenario 1, we drew $\beta _{g3}$ values from a gamma distribution with a mode of log(2) and a standard deviation of 0.5 that were then randomly assigned to be positive or negative; in Simulation Scenario 2, we randomly assigned $\beta _{g3}$ to either 0.375 or − 0.375. This created a change in expression over time for subjects in the treatment group for 20% of genes.

Simulated data for both scenarios were modeled using DREAM, lmerSeq using random effects, lmerSeq using covariance structures, and rmRNAseq. In Scenario 1, all models were fit with the same fixed effects (group, time and a group-by-time interaction). Both DREAM and lmerSeq (RI) were fit using a random intercept, while lmerSeq (CS) utilized a compound symmetric residual correlation structure instead of random effects. Even though rmRNAseq only allows a CAR structure, with only two observations separated by a single unit of time this is equivalent to a compound symmetric structure. Therefore, all of the methods were fit with with a correctly specified model in Scenario 1.

In Scenario 2, we fit a variety of models using different combinations of fixed and random effects to compare the methods’ performance under the correct model specification, misspecified random effects or covariance structures, and different fixed effects modeling strategies. We considered two fixed effects modeling strategies: one using a continuous time predictor and its interaction with the binary group variable, and another using categorical time and its interaction with group. While both models are correctly specified, the first model assumes linear changes in expression over time. The second approach does not make assumptions about the pattern of change over time, but uses additional degrees of freedom due to the inclusion of multiple time point indicator variables and their associated group interaction terms in the regression model. For rmRNAseq, only the CAR correlation structure is available, though this model is supposed to be robust to misspecification since an unstructured covariance matrix is used for some portions of the model fitting and testing algorithm [11]. With DREAM and lmerSeq, we considered models including only a random intercept and models including both a random intercept and random slope. For lmerSeq, we also fit models using categorical time fixed effects and an unstructured covariance matrix as a flexible alternative to including random effects.

We evaluated type 1 error rates, power (sensitivity) and False Discovery Rates (FDRs) for three statistical tests: a between-subject contrast (difference between groups at the last follow-up visit), a within-subject contrast (change over time in the treatment group), and an interaction test (difference in change over time between the two groups). Inference for DREAM and lmerSeq was based on t-tests using Satterthwaite degrees of freedom. Adjusted *p* values were obtained using the Benjamini–Hochberg method, and these were used for calculating observed FDRs and sensitivities at various thresholds [21]. rmRNAseq calculates *p* values based on a bootstrap approach (we used 100 iterations as suggested in the package vignette) after calculating moderated F-statistics, and then q-values for FDR control based on the methods of [22, 23]. However, we applied the Benjamini–Hochberg method to the raw *p* values returned by rmRNAseq to ensure that any differences observed between this method and the others could not be attributed to using an alternative strategy for multiple comparisons adjustments.

### Case study

To assess the performance of these analysis methods in a realistic setting, we analyzed a publicly available dataset (GEO Dataset: GSE131411) including RNA-Seq of whole blood from 11 cardiogenic shock patients at three time points: (1) within 16 h of intensive care unit admission, (2) 48 h after admission, and (3) 7 days after admission or at discharge. This study has been fully described in Braga et al. [4]. We used lmerSeq, DREAM, and rmRNAseq to compare gene expression between the three time points. We limited our analysis to 13,123 genes with at least 1 count per million reads in 11 of the 33 samples. For lmerSeq and DREAM, a random intercept was used to account for correlation between repeated measures, and categorical time was the only fixed effect included in the models. All other aspects of the analysis were performed as described for Simulation Scenario 1 using the default settings for each method. Raw *p* values were adjusted using the Benjamini–Hochberg method to control the FDR.

To further understand the statistical testing properties of these methods in a more realistic setting, we also utilized this data to perform simulations using a permutation method. This allowed for simulated datasets that could be generated with characteristics of real RNA-Seq data without relying on distributional assumptions for the underlying count data. In addition, these data have an unknown correlation structure between repeated measurements on the same subject. To create a simulated dataset, ten subjects were randomly selected for inclusion. For five of the subjects, the labels were switched for the baseline and 1 week follow up time points, so there would be no expected differential expression between these two time points. Then, for 2600 randomly selected genes, differential expression was created by multiplying the counts at the 1 week follow up by either 2 (50%) or 0.5 (other 50%). Ten simulated datasets were created and analyzed as described above for the formal analysis of the original data, and FDR and power were calculated across the 10 simulations and averaged for each method.

## Results

### Run times

Table 1 has the average run times for the correctly specified version of each method at each sample size in Simulation Scenario 2. These times include all of the steps needed to go from the raw RNA-Seq counts to the transformed values used for analysis, and all the way through generating the final summary tables. Where possible, parallelization was utilized for each method with 4 cores being allocated for computations. In all scenarios, lmerSeq was the fastest method. The increased computation time for DREAM was largely due to the time needed to calculate the precision weights for their modified VOOM transformation which also requires the fitting of LMMs for each gene; the VST used for lmerSeq generally only took a few seconds. The rmRNAseq runtimes were orders of magnitude longer than either lmerSeq or DREAM due to the reliance on re-sampling for calculating *p* values with the smallest datasets taking over 7 h to analyze compared to about 6 and 10 min for lmerSeq and DREAM respectively.

**Table 1:** Runtimes in minutes at all sample sizes for the correctly specified version of each method in Simulation 2

N per group	lmerSeq	DREAM	rmRNAseq
3	5.58	10.72	426.14
5	5.60	11.03	455.76
10	5.68	11.30	602.53
20	6.08	11.50	751.97

### Error rates and sensitivity

**Fig. 1:**
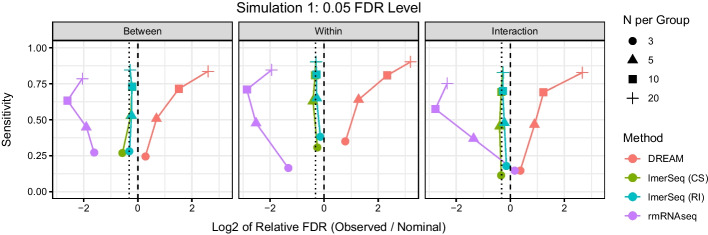
Scatter plot of sensitivity by log$_2$ of the relative false discovery rate (FDR) for each type of test at each sample size at the 0.05 level in Simulation 1. The dashed vertical line represents the nominal rate, while the dotted vertical line represents the expected FDR. *RI* random intercept, *CS* compound symmetric covariance matrix

For Simulation Scenario 1 with two repeated measures per subject, the relationship between FDR control and sensitivity at the 0.05 FDR level is presented in Fig. 1 (see Additional file 1: Figs. S1–S2 for the 0.01 and 0.10 FDR levels). In this scenario, DREAM, lmerSeq and rmRNAseq models are all correctly specified. DREAM has slightly inflated FDRs at $N=3$, with FDR inflation becoming substantially worse with increasing sample size. In contrast, rmRNAseq is highly conservative at almost all sample sizes and is typically the least sensitive method. lmerSeq is always slightly below the nominal FDR with values converging to the expected FDR as sample size increases. Moreover, lmerSeq is almost uniformly more powerful than rmRNAseq and often achieves higher power than DREAM, despite the fact that DREAM has inflated FDRs. The two versions of lmerSeq, which are modeling the same covariance structure in slightly different ways, offer nearly identical results with minor inconsistencies likely due to differences in numerical estimation methods in the nlme and lme4 R packages.

**Fig. 2:**
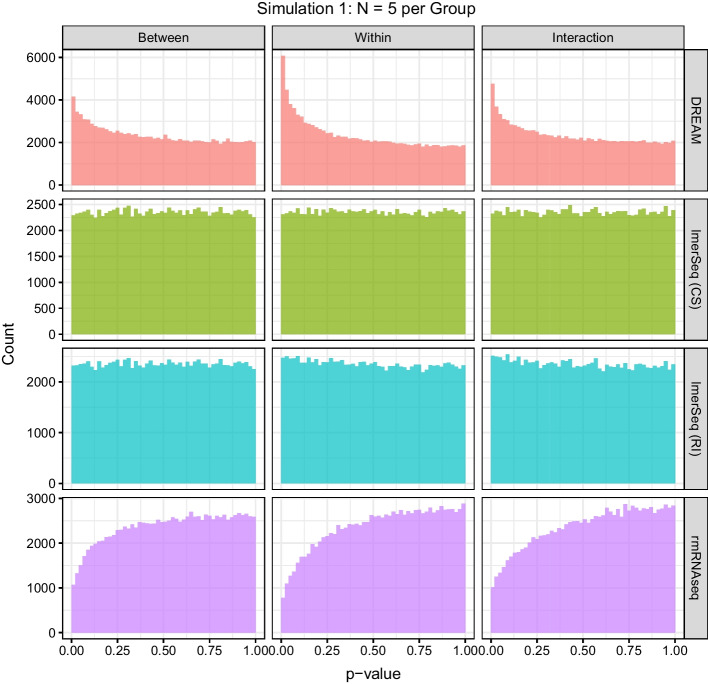
Histograms of the *p* values for the null features across all datasets in Simulation 1 with $N=5$ subjects per group. *RI* random intercept, *CS* compound symmetric covariance matrix

The distribution of the *p* values from all of the “null” genes for each of the three tests are displayed for the $N=5$ simulations in Fig. 2 (other sample size are shown in Additional file 1: Figs. S3–S5). Under the null hypothesis, well calibrated *p* values for these features should follow a uniform distribution; however, DREAM exhibits an overabundance of *p* values close to zero, corresponding to the inflated error rates. Again, this problem becomes worse with increasing sample size. For rmRNAseq, we see the opposite behavior, with fewer small *p* values than expected, corresponding to the overly conservative observed error rates and lower sensitivity than the other methods. Conversely, the distributions for both versions of lmerSeq are nearly uniform by $N=5$.

**Fig. 3:**
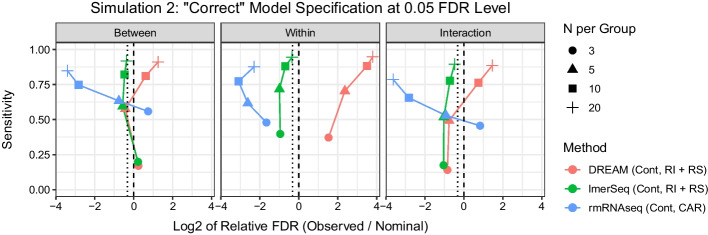
Scatter plot of sensitivity by log$_2$ of the relative false discovery rate (FDR) for each type of test at each sample size at the 0.05 level for the models with correct (or as close as possible given method constraints) specification of both the fixed and random effects in Simulation 2. The dashed vertical line represents the nominal rate, while the dotted vertical line represents the expected FDR. *Cont* continuous time, *RI* random intercept, *RS* random slope, *CAR* continuous auto regressive

**Fig. 4:**
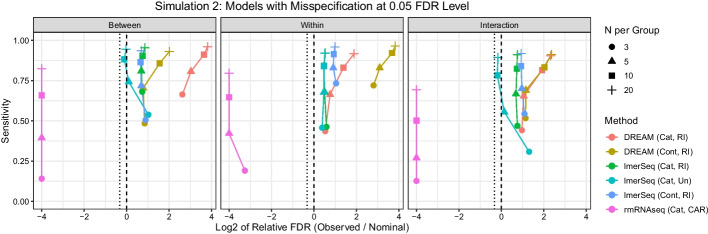
Scatter plot of sensitivity by log$_2$ of the relative false discovery rate (FDR) for each type of test at each sample size at the 0.05 level for the models with some misspecification of fixed and/or random effects in Simulation 2. The dashed vertical line represents the nominal rate, while the dotted vertical line represents the expected FDR. *Cont* continuous time, *Cat* categorical time, *RI* random intercept, *RS* random slope, *UN* unstructured covariance matrix, *CAR* continuous auto regressive covariance matrix

In the second simulation scenario with four repeated measures per subject and both a random intercept and slope, we are able to consider the impact of misspecification of the random effect or correlation structure, as well as different fixed effects modeling strategies. First we consider models where time is modeled as a continuous variable. In models with correctly specified random effects structure (Fig. 3 and Additional file 1: Figs. S6–S8), including both a random intercept and slope, performance was similar to Simulation Scenario 1: lmerSeq approached the expected FDR with increasing sample size, while DREAM showed greater FDR inflation with increasing sample size. In models with a misspecified random effects structure (including a random intercept only), FDR is increased relative to the models with correctly specified random effects for both lmerSeq and DREAM, consistent with previous findings that random effects misspecification can result in increased numbers of false positives (Fig. 4 and Additional file 1: Figs. S6–S8) [24–26]. rmRNAseq, which uses a CAR covariance structure, remains overly conservative with noticeably lower sensitivity in most scenarios, although FDR inflation is seen for between-subject and interaction tests at the smallest sample size ($N=3$ per group).

Next we considered models coding time as a categorical factor, represented by three indicator variables, which allows gene expression to change flexibly over time (Fig. 4 and Additional file 1: Figs. S6–S8). This approach is likely to be used in practice as the assumption of linear changes in gene expression over time often may not be reasonable. rmRNAseq’s FDRs remain very conservative for all tests, resulting in reduced power to detect associations. For DREAM and lmerSeq using just a random intercept, FDRs are inflated relative to a model with correctly specified random effects structure for between and interaction tests for DREAM and for all three tests for lmerSeq. For DREAM, FDRs were similar to continuous time models with a random intercept only for interaction tests, higher for between subject tests, and lower for within subject tests. For lmerSeq, FDRs were similar or smaller compared to the continuous time models with a random intercept only. For lmerSeq, we also considered a completely flexible model using categorical time and an unstructured covariance matrix. This model has several more parameters to estimate compared to a model using continuous time and a random intercept and slope (4 additional regression coefficients + 8 additional covariance parameters). For small sample sizes ($N=3$), FDRs are above their nominal levels, likely due to over-fitting of the data; however, as sample size increases, FDR converges towards the nominal rate. This suggests that if there are sufficient numbers of subjects, this flexible approach may help safeguard against false positives due to model misspecification.

**Fig. 5:**
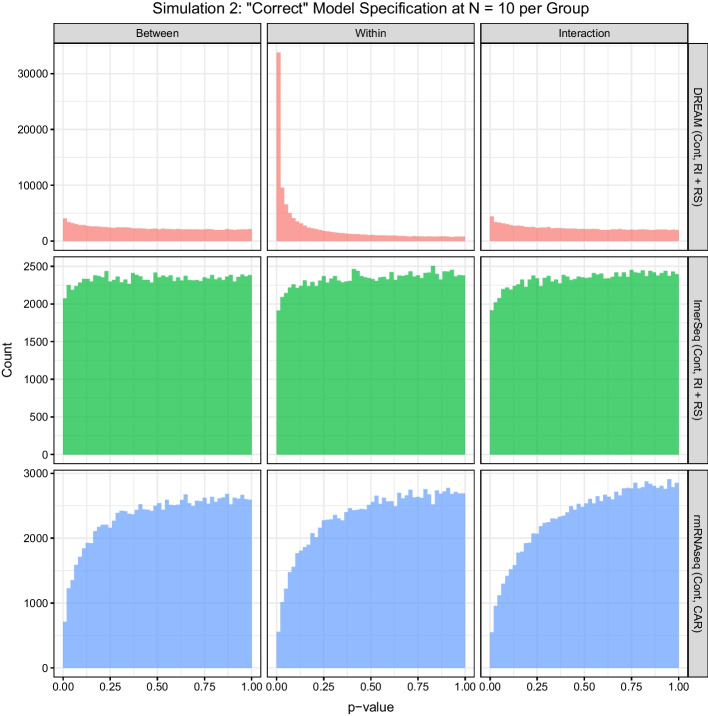
Histograms of the *p* values for the null features across all datasets in Simulation 2 for the models with the most correct specification of fixed and random effects at $N=10$ subjects per group. For both DREAM and lmerSeq the fixed and random effects structures were able to exactly match the simulated data, while rmRNAseq could only match the correct fixed effects since it only offers CAR for modeling correlation between observations. *Cont* continuous time, *RI* random intercept, *RS* random slope, *CAR* continuous auto regressive

The *p* value histograms for the truly “null” features in Simulation Scenario 2 are presented for $N=10$ in Fig. 5 and Additional file 1: Fig. S9 with other sample sizes presented in Additional file 1: Figs. S10–S12. As in Simulation Scenario 1, lmerSeq with the proper specification of fixed and random effects has nearly uniform distributions for each contrast. DREAM and rmRNAseq have significant right and left skew to their respective distributions, corresponding to the observed error rate control with DREAM having inflated FDRs and rmRNAseq being conservative. Since there was no correlation between the counts for any two genes in the simulated data, all of the tests within a given contrast are independent. Consequently, the divergences from uniformity in the *p* value histograms for both DREAM and rmRNAseq suggest that the assumed distributions for the test statistics are incorrect, and thus the resulting *p* values are not reliable [27].

### Case study

Table 2 shows a comparison the results of the analyses from each method. An advantage of lmerSeq is that it automatically reports and excludes singularities from tables of results and full model fits are returned, so a variety of existing R packages can be used to assess model fit. In this analysis, a small percentage of the lmerSeq model fits were singular (1.5%) and were excluded from the results. Since DREAM does not do any such reporting and exclusion of genes with singular fits, we refit all of the models using lmerSeq with weights to analyze the VOOM transformed data and found that almost 100 singular fits were also included in the DREAM results. To evaluate whether modeling assumptions were met, we used Levene’s method to test for equality of variance between time points and a Kolmogorov-Smirnov test for normality of scaled residuals. For lmerSeq, 1.6% of genes had unadjusted Levine test *p* values less than 0.05, compared to 1.1% for DREAM; after taking a multiple comparisons adjustment, none of the genes showed significant heteroskedasticity in the residuals for either method. Less than 0.2% of models had Kolmogorov–Smirnov test *p* values less than 0.05; after adjusting for multiple comparisons none of the models showed significant deviations from normality. None of the preceding diagnostics are possible with rmRNAseq.

**Table 2:** Summary of model diagnostics and differential expression for each method when applied to the real RNA-Seq data from the case study

	lmerSeq	DREAM	rmRNAseq
Singular fits	200 (1.5%)	96 (0.7%)*	Not available
Levene’s test for heteroskedsticity			
*p* vaule $<0.05$	206 (1.6%)	149 (1.1%)	Not available
BH adjusted *p* value $<0.05$	0	0	Not available
Kolmogorov–Smirnov test for normality			
*p* value $<0.05$	24 (0.2%)	17 (0.1%)	Not available
BH adjusted *p* value $<0.05$	0	0	Not available
Number of DEGs			
48 h versus baseline	1	0	0
1 week versus baseline	1452	1828	142
1 week versus 48 h	362	476	182

*BH* Benjamini–Hochberg, *DEGs* differentially expressed genes

*Models had to be refit with lmerSeq using weights since DREAM does not report or exclude singular fits

In terms of the number of differentially expressed genes (DEGs), results were largely consistent with what would be expected based on our simulation studies. For brevity, we will focus on the differences in expression between baseline and the 1 week follow up, which had the largest number of DEGs. rmRNAseq found relatively few DEGs compared to the other two methods, aligning with the overly conservative behavior noted in our simulation study, while DREAM, which had inflated false discovery rates, found the largest number of DEGs. We performed functional enrichment analysis on the up and down regulated genes from lmerSeq using enrichR and the BioPlanet 2019 database [28, 29]. Genes upregulated at 1 week compared to baseline were enriched for pathways related to cell cycle and the complement cascade, while down regulated genes were enriched for pathways related to innate immunity and inflammation.

Results for the permutation-based simulation study using the real RNA-Seq data are presented in Table 3 when using a 5% FDR threshold to identify differential expression. Similar to our other simulation studies, DREAM had inflated FDRs (more than double the nominal rate), while lmerSeq maintained the nominal 5% FDR while also having higher power than DREAM. rmRNAseq had a slightly conservative FDR and lower power than the other methods in this simulation.

**Table 3:** Summary of the observed false discovery rates (FDR) and power from the permutation based simulations using the real RNA-Seq data from the case study

Method	FDR	Power
lmerSeq	0.051	0.912
DREAM	0.114	0.900
rmRNAseq	0.036	0.883

Values presented are the means taken across the 10 simulated datasets when using a nominal FDR of 0.05 to identify differential expression

## Conclusions

Accounting for repeated measures in RNA-Seq studies by transforming the digital counts and then fitting normal LMMs is an appealing analysis strategy due to the well-established theoretical work surrounding LMMs and their computational efficiency compared to GLMMs. However, the selection of the transformation, modeling strategy, and the type of statistical test used can drastically alter the results. Both DREAM and rmRNAseq utilize the VOOM transformation, but fit LMMs in different ways and use different strategies to compute test statistics and *p* values. This leads to divergent behavior in our simulation studies, with DREAM exhibiting substantial FDR inflation and rmRNAseq being overly conservative. Though rmRNAseq did show improvement going from $N=10$ to $N=20$ in some scenarios, both methods tended to perform worse in terms of FDR control with increasing sample size, which is concerning. Since DREAM and lmerSeq are capable of fitting similar LMMs and we fit many of the same model structures with both methods, it appears that the driving force behind the differential behavior between lmerSeq and DREAM is the choice of transformation, with lmerSeq utilizing DESeq2’s VST and DREAM using their own modification of VOOM. Interestingly, the VST has also shown superior performance compared to VOOM when estimating heritability for sequencing data, with those methods also being based on linear mixed model fits [15]. Regardless of the causes for the differences in behavior between the methods, we found lmerSeq had the best control of FDRs (i.e. closest to nominal and expected levels), generally had the highest sensitivity, and had the shortest run times. This was evident over multiple simulation scenarios with some being based on common distributional assumptions used in RNA-Seq analysis, and others based solely on real RNA-Seq data from a study with repeated measures where no additional assumptions about distributions or covariance structures were made. Moreover, our case study results also align with the general behavior observed in the simulations studies. The lmerSeq R package offers more flexibility than either DREAM or rmRNAseq in terms of allowing for multiple random effects and a variety of correlation structures, which can help guard against false positives due to model misspecification. Additionally, lmerSeq allows for greater scrutiny of modeling assumptions by identifying singular fits and gives the user the ability to test for heteroskedasticity, normality, or any other diagnostics available for nlme or lme4 objects. Ultimately, the totality of our results suggest that using lmerSeq in conjunction with the VST is currently the most complete and reliable source of inference for analyzing transformed bulk RNA-Seq data from studies that require the analyst to account for correlation between observations.

## Supplementary Information

**Additional file 1: Supplementary figures** S1–S12.

## Data Availability

Simulated datasets and the code used to produce the results presented in this paper are available at https://github.com/stop-pre16/lmerSeq-paper-data-and-code

## Abbreviations

CAR: Continuous autoregressive
CS: Compound symmetric
DEGs: Differentially expressed genes
FDR: False discovery rate
GLMM: Generalized linear mixed model
LMM: Linear mixed model
RI: Random ntercept
RNA-Seq: RNA sequencing
VST: Variance stabilizing transformation
