# Supplementary material for: lmerSeq: an R package for analyzing transformed RNA-Seq data with linear mixed effects models
Source: BMC Bioinformatics. 2022 Nov 16;23:489. doi: 10.1186/s12859-022-05019-9 (PMC9670578; doi:10.1186/s12859-022-05019-9)

lmerSeq: an R package for analyzing transformed RNA-Seq data with linear mixed effects models: Additional File 1

Brian E. Vestal, Elizabeth Wynn, Camille M. Moore

1 Supplementary Figures

Figure S1: Scatter plot of sensitivity by $\log_2$ of relative False Discovery Rate (FDR) for each type of test at each sample size at the 0.01 level in Simulation 1. The dashed vertical line represents the nominal rate, while the dotted vertical line represents the expected FDR.

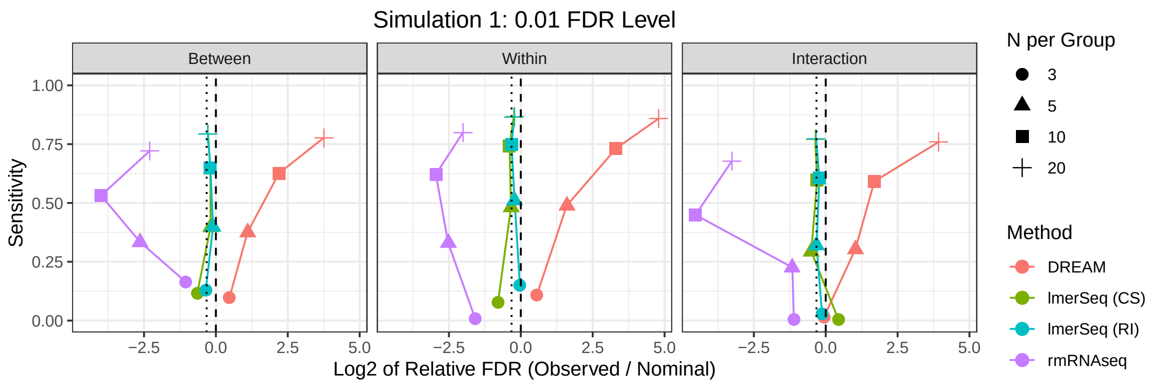

Figure S2: Scatter plot of sensitivity by $\log_2$ of relative False Discovery Rate (FDR) for each type of test at each sample size at the 0.10 level in Simulation 1. The dashed vertical line represents the nominal rate, while the dotted vertical line represents the expected FDR.

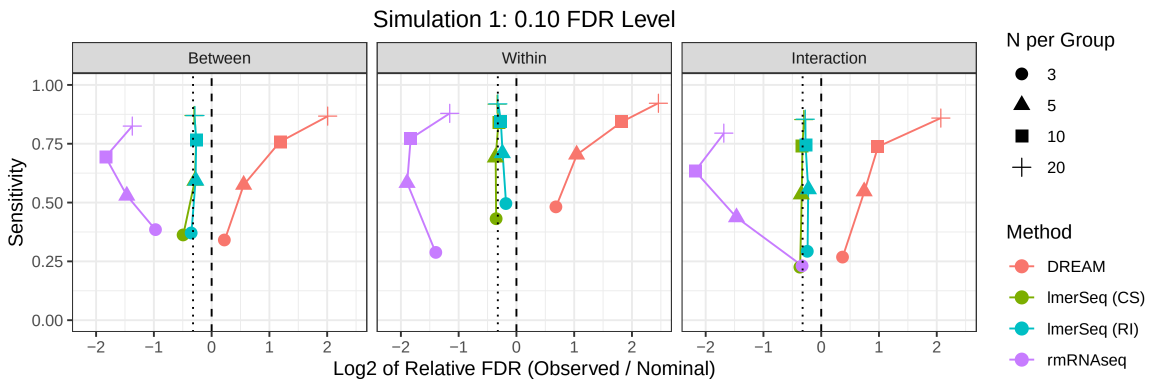

Figure S3: Histograms of the p-values for the null features across all datasets in Simulation 1 with $N = 3$ subjects per group.

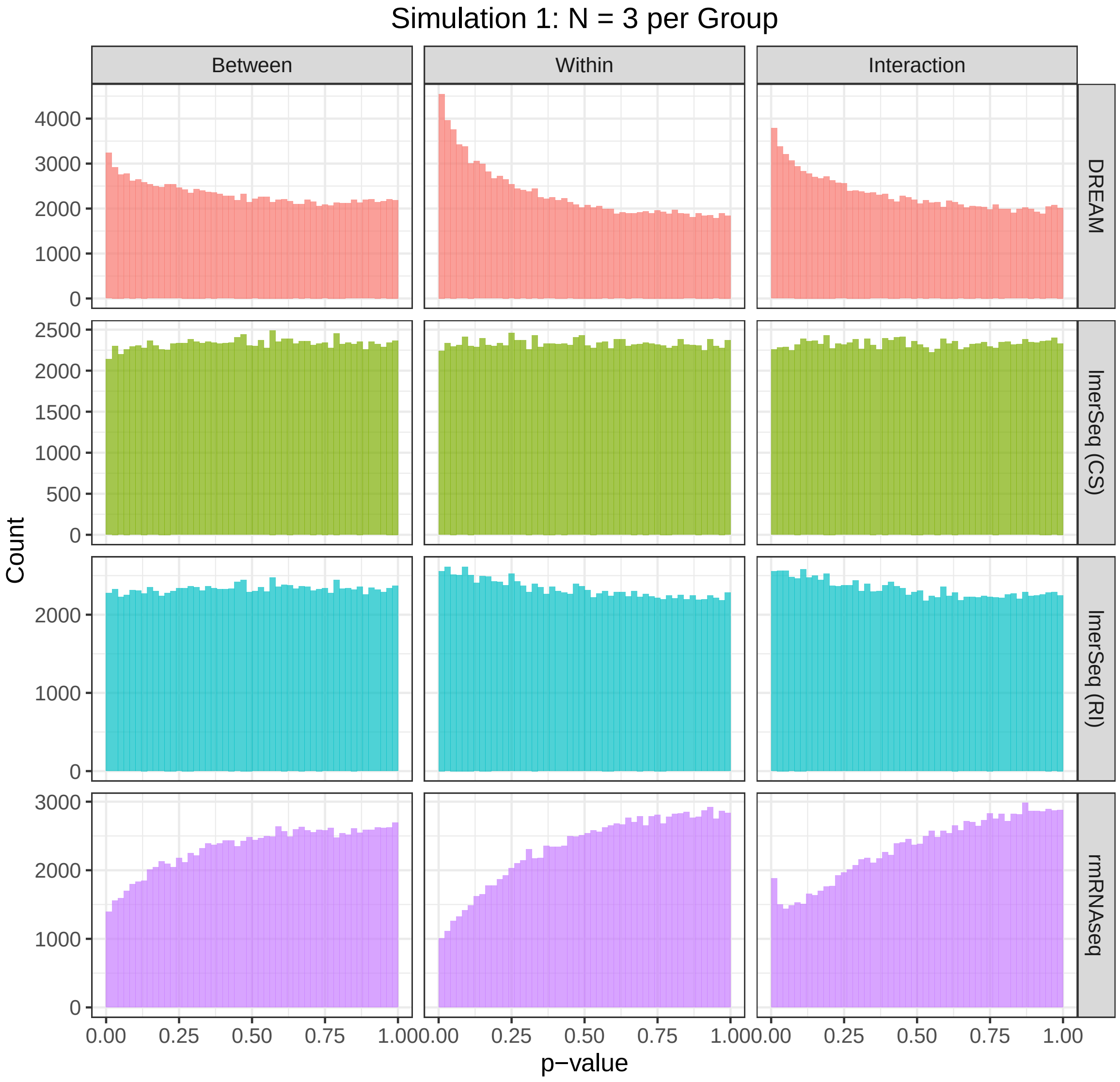

Figure S4: Histograms of the p-values for the null features across all datasets in Simulation 1 with $N = 10$ subjects per group.

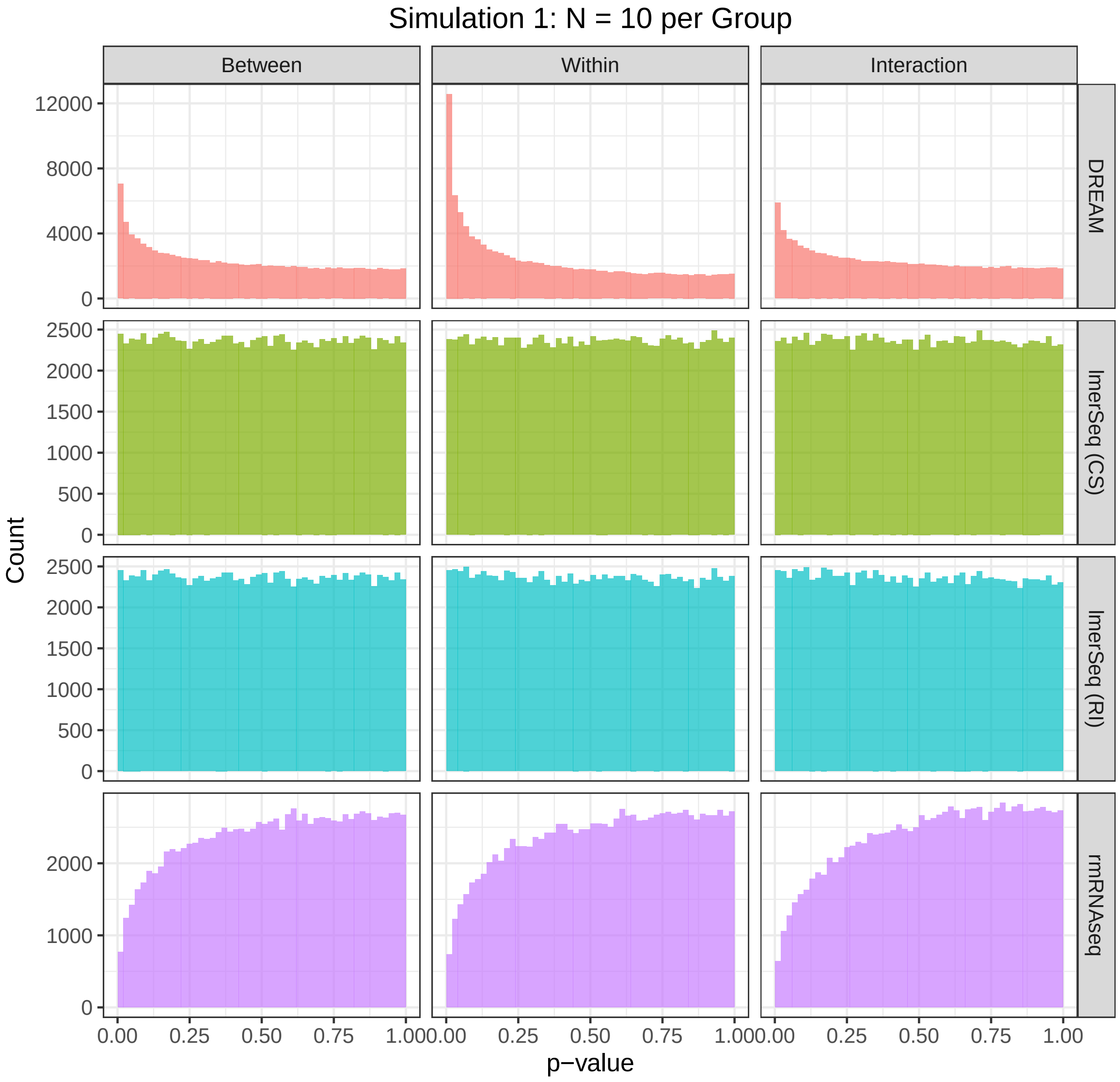

Figure S5: Histograms of the p-values for the null features across all datasets in Simulation 1 with $N = 20$ subjects per group.

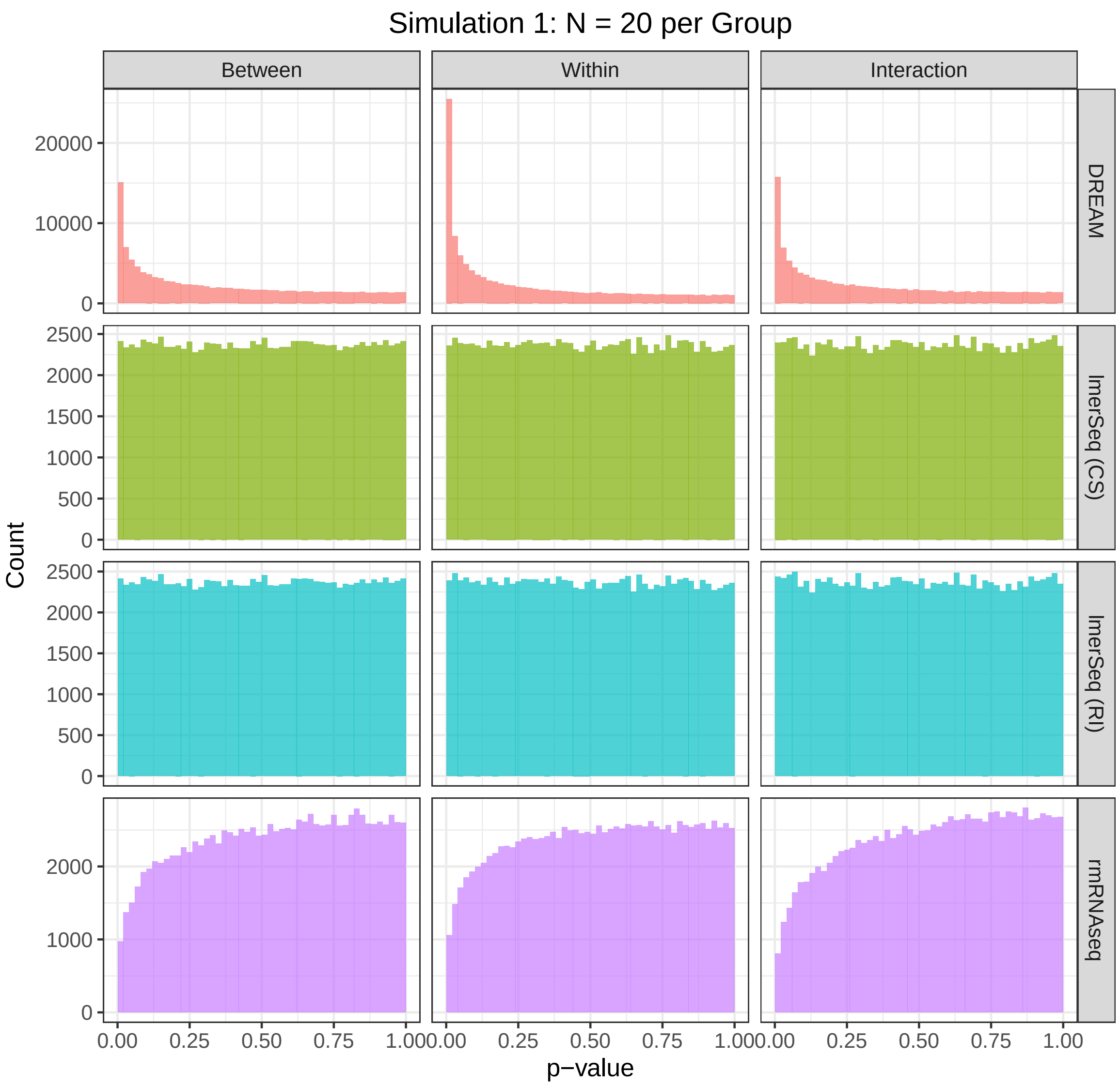

Figure S6: Scatter plot of sensitivity by $\log_2$ of relative False Discovery Rate (FDR) for each type of test at each sample size at the 0.01 level in Simulation 2. The dashed vertical line represents the nominal rate, while the dotted vertical line represents the expected FDR. Cont = continuous time, Cat = categorical time, RI = random intercept, RS = random slope, UN = unstructured covariance matrix, CAR = continuous auto regressive

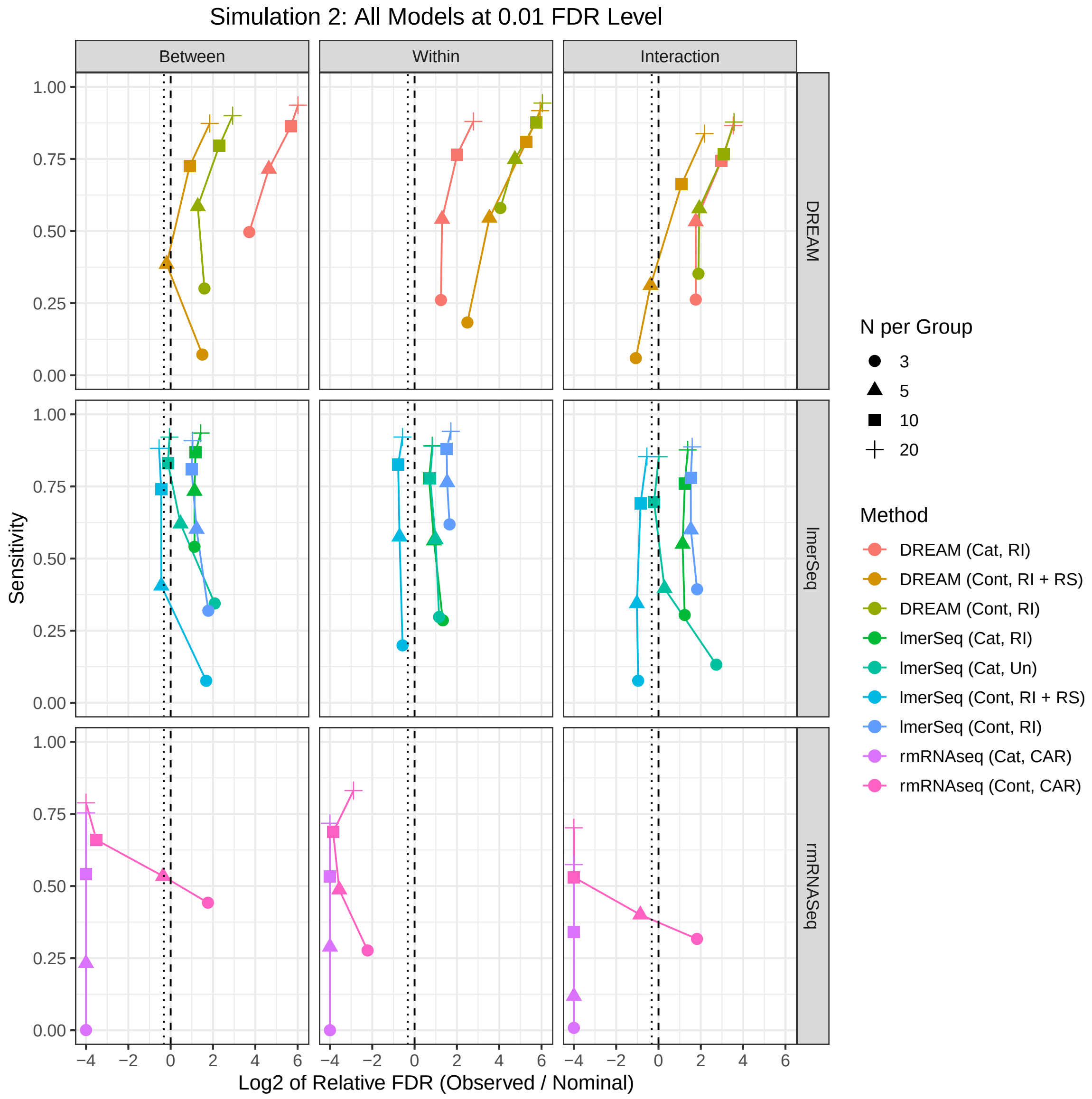

Figure S7: Scatter plot of sensitivity by $\log_2$ of relative False Discovery Rate (FDR) for each type of test at each sample size at the 0.05 level in Simulation 2. The dashed vertical line represents the nominal rate, while the dotted vertical line represents the expected FDR. Cont = continuous time, Cat = categorical time, RI = random intercept, RS = random slope, UN = unstructured covariance matrix, CAR = continuous auto regressive

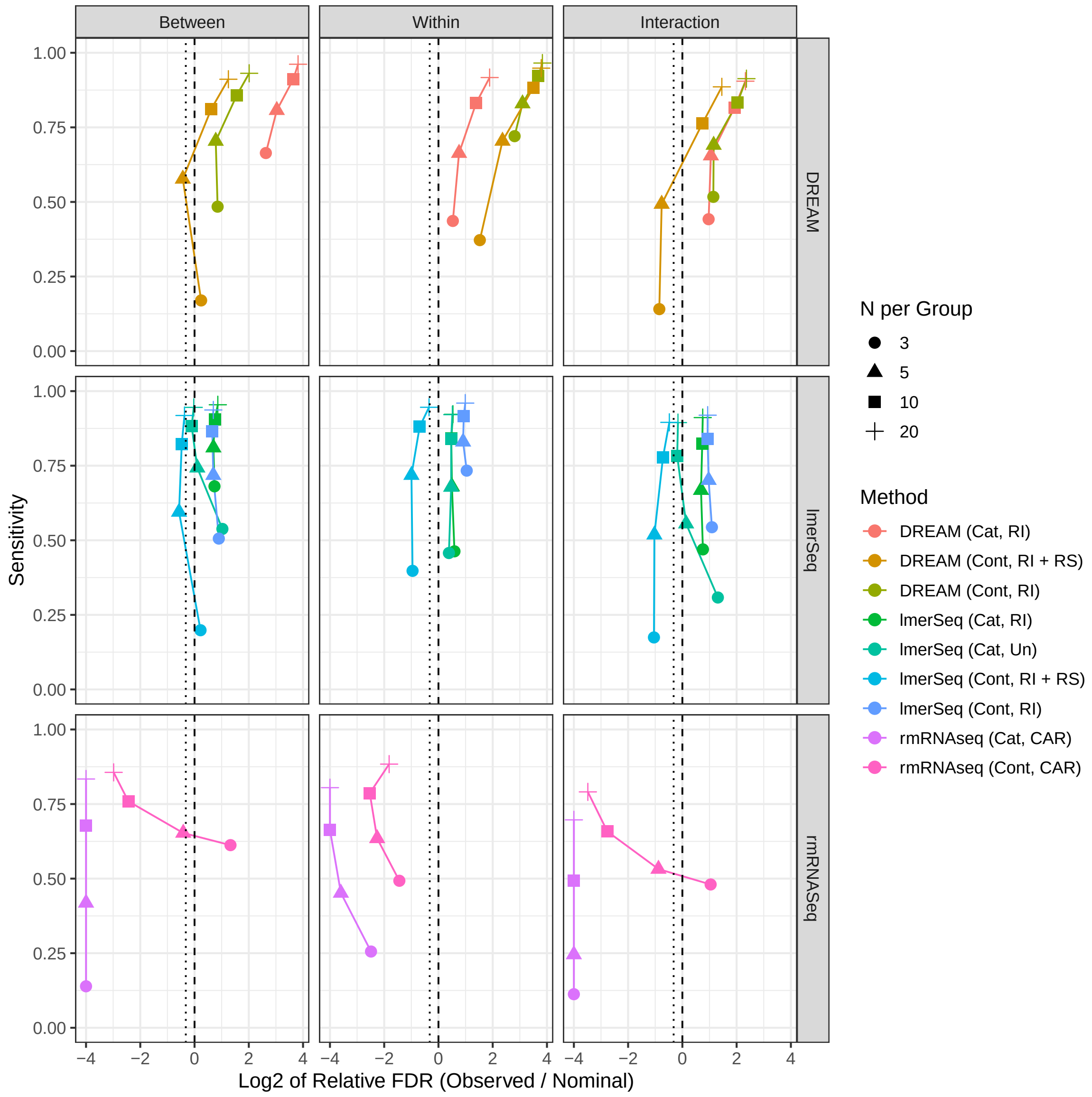

Figure S8: Scatter plot of sensitivity by $\log_2$ of relative False Discovery Rate (FDR) for each type of test at each sample size at the 0.10 level in Simulation 2. The dashed vertical line represents the nominal rate, while the dotted vertical line represents the expected FDR. Cont = continuous time, Cat = categorical time, RI = random intercept, RS = random slope, UN = unstructured covariance matrix, CAR = continuous auto regressive

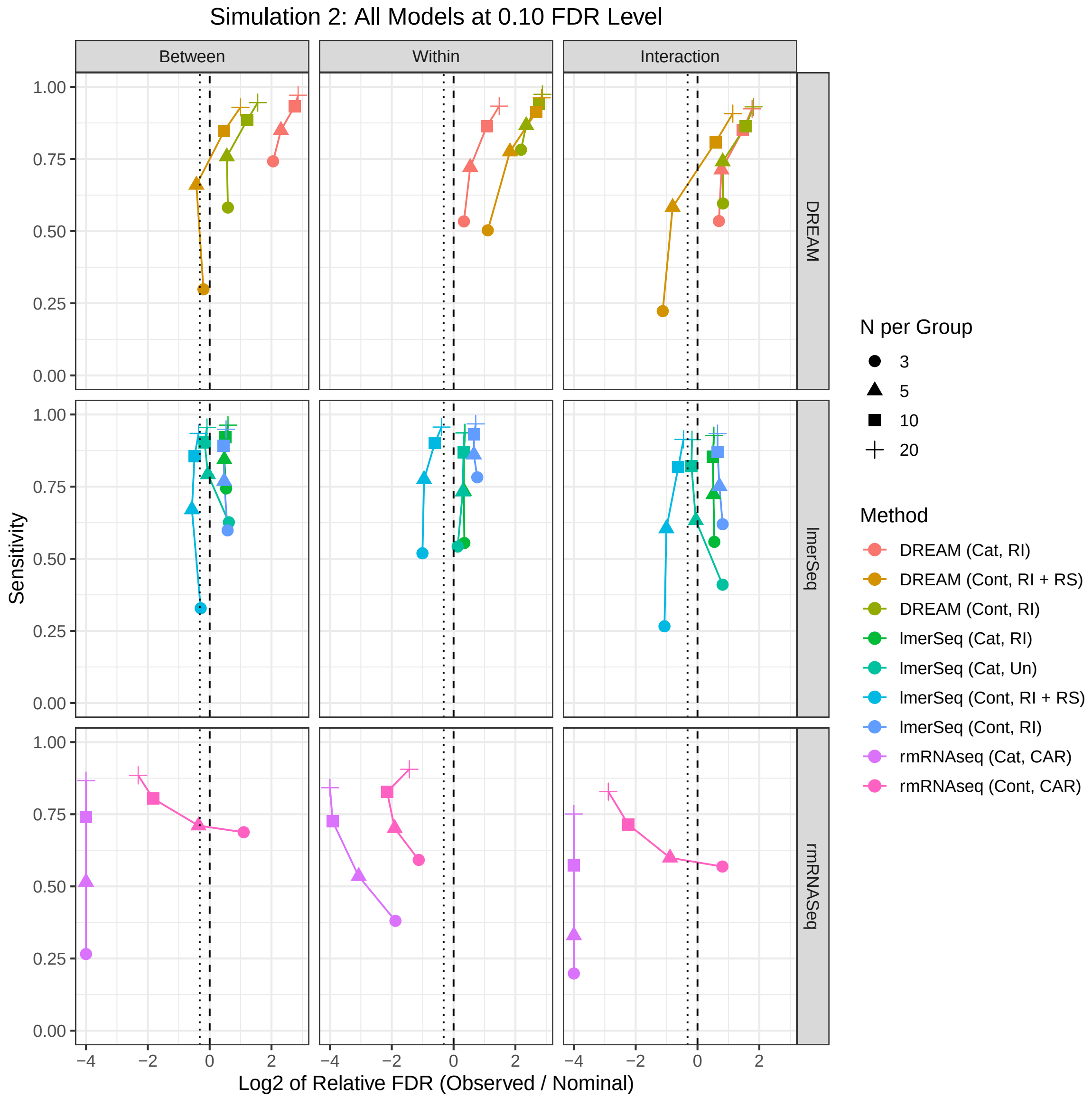

Figure S9: Histograms of the p-values for the null features across all datasets in Simulation 2 with $N = 10$ subjects per group. Cont = continuous time, Cat = categorical time, RI = random intercept, RS = random slope, UN = unstructured covariance matrix, CAR = continuous auto regressive

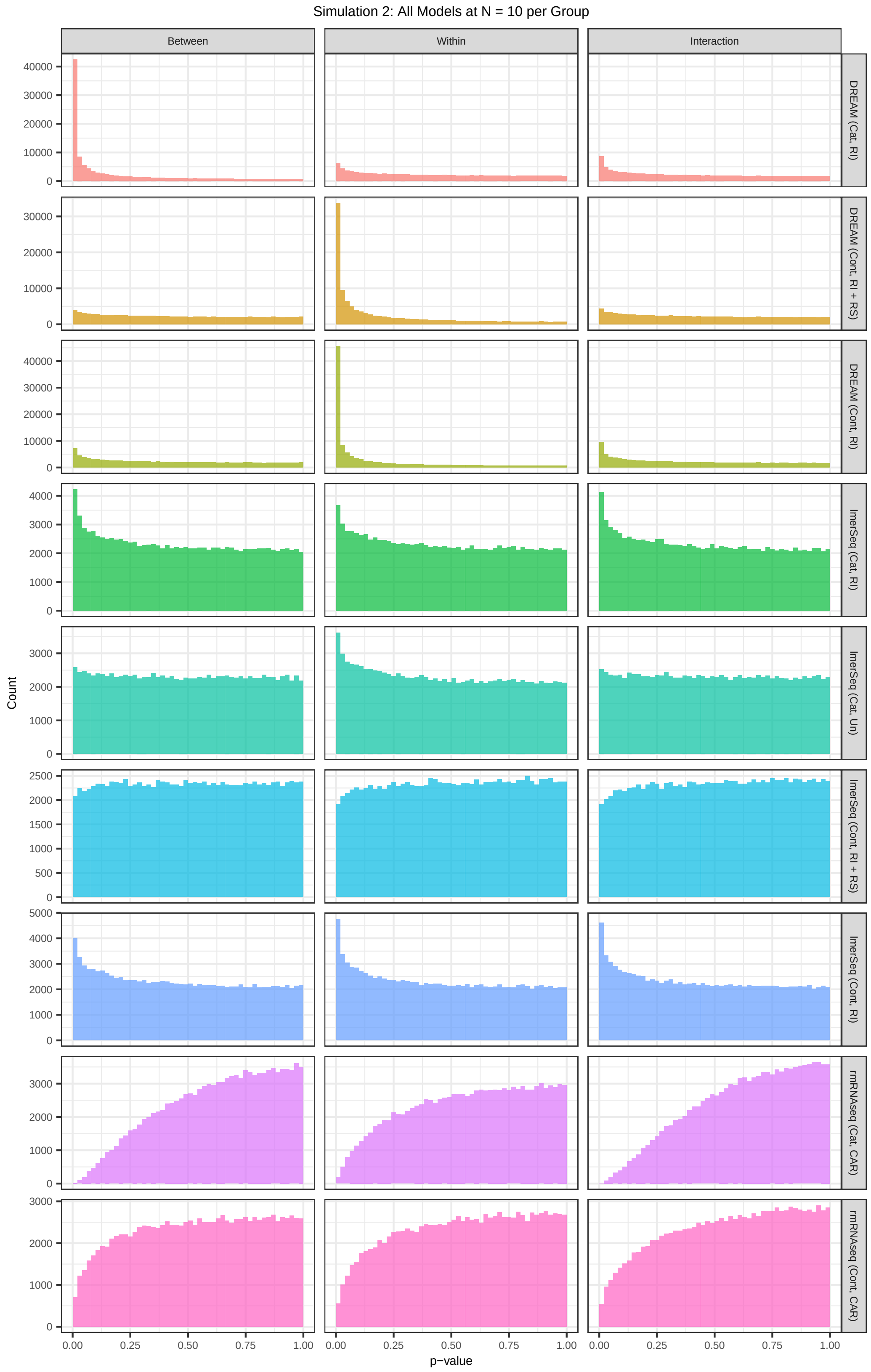

Figure S10: Histograms of the p-values for the null features across all datasets in Simulation 2 with $N = 3$ subjects per group. Cont = continuous time, Cat = categorical time, RI = random intercept, RS = random slope, UN = unstructured covariance matrix, CAR = continuous auto regressive

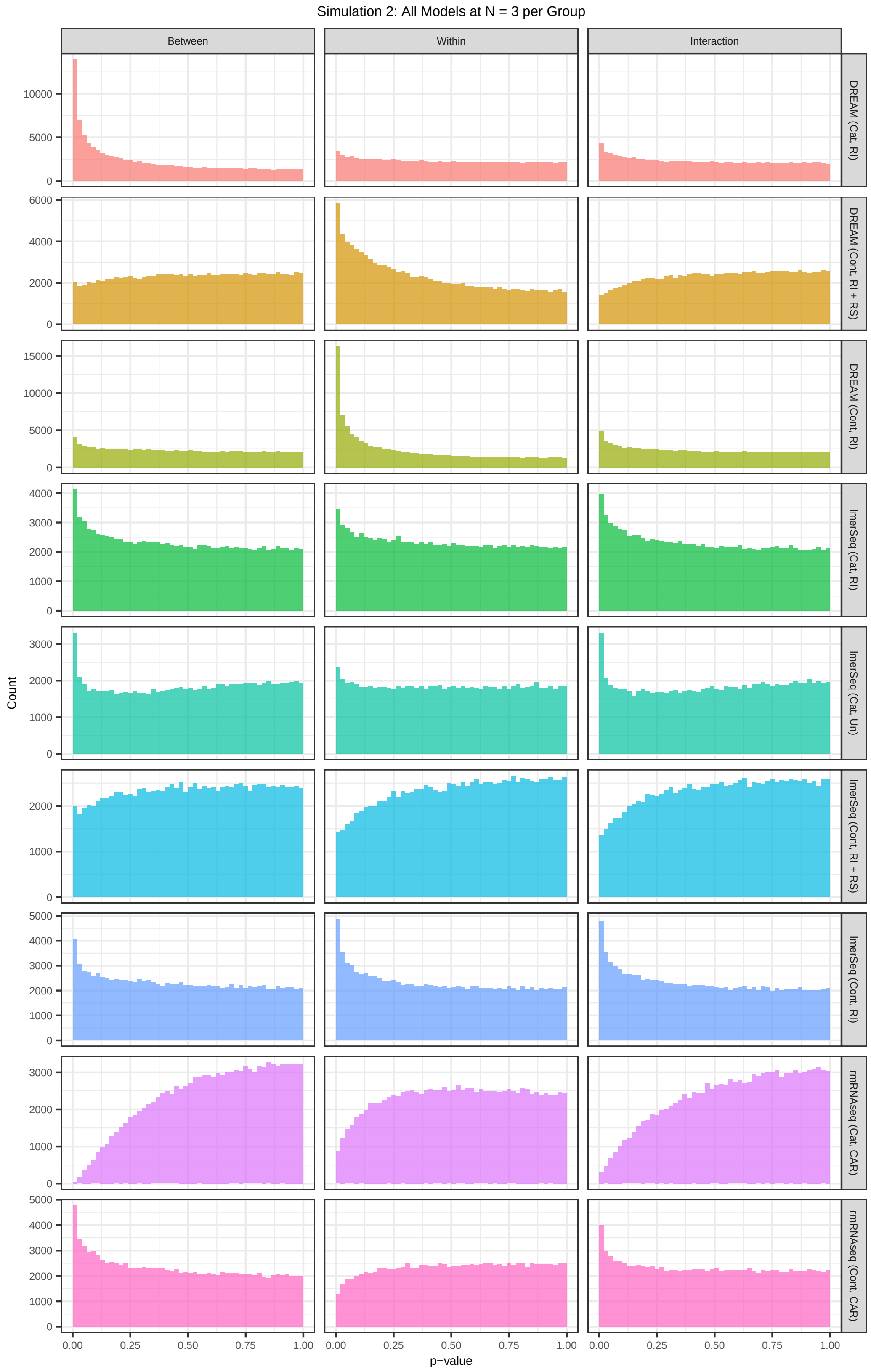

Figure S11: Histograms of the p-values for the null features across all datasets in Simulation 2 with $N = 5$ subjects per group. Cont = continuous time, Cat = categorical time, RI = random intercept, RS = random slope, UN = unstructured covariance matrix, CAR = continuous auto regressive

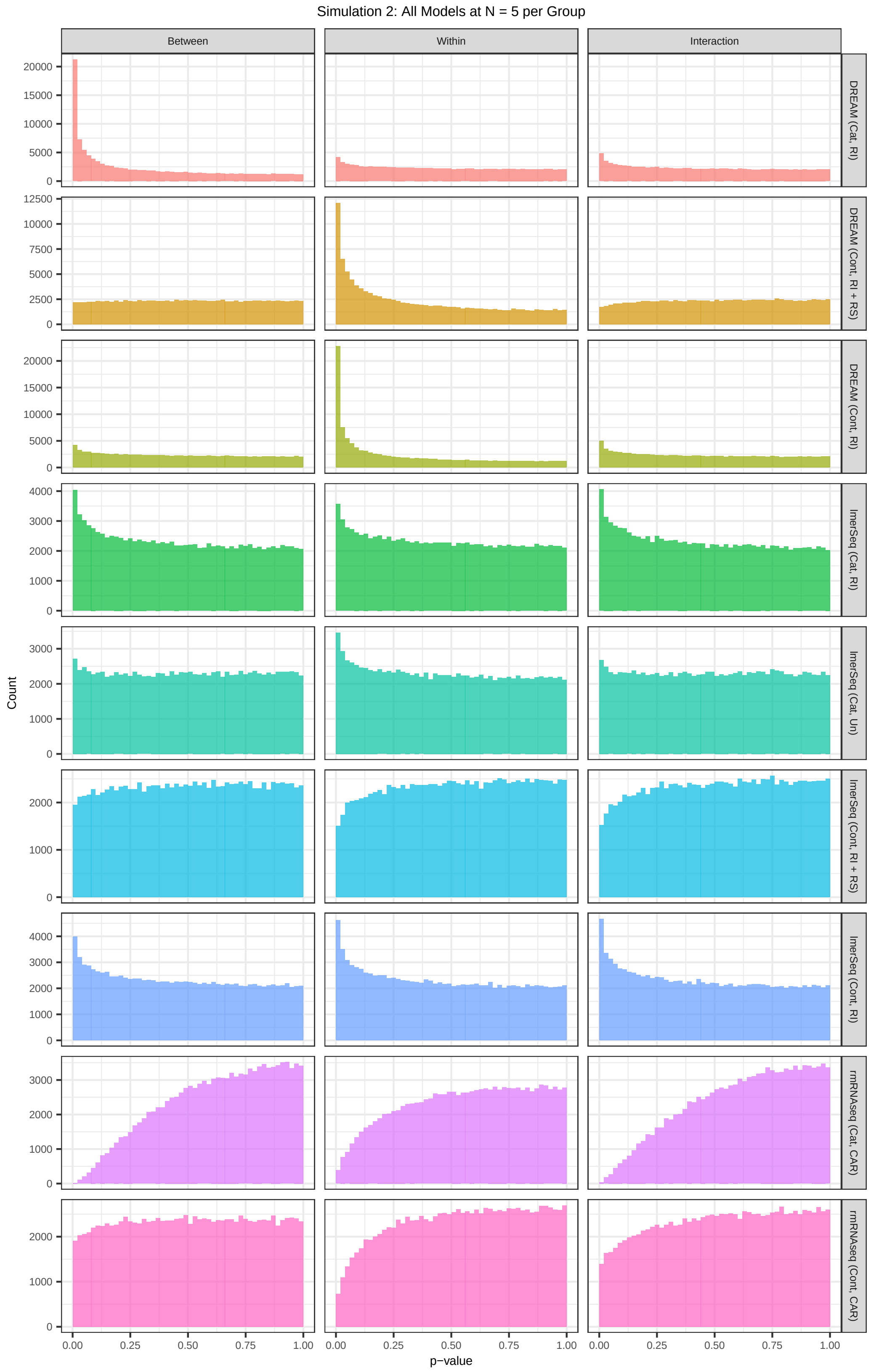

Figure S12: Histograms of the p-values for the null features across all datasets in Simulation 2 with $N = 20$ subjects per group. Cont = continuous time, Cat = categorical time, RI = random intercept, RS = random slope, UN = unstructured covariance matrix, CAR = continuous auto regressive

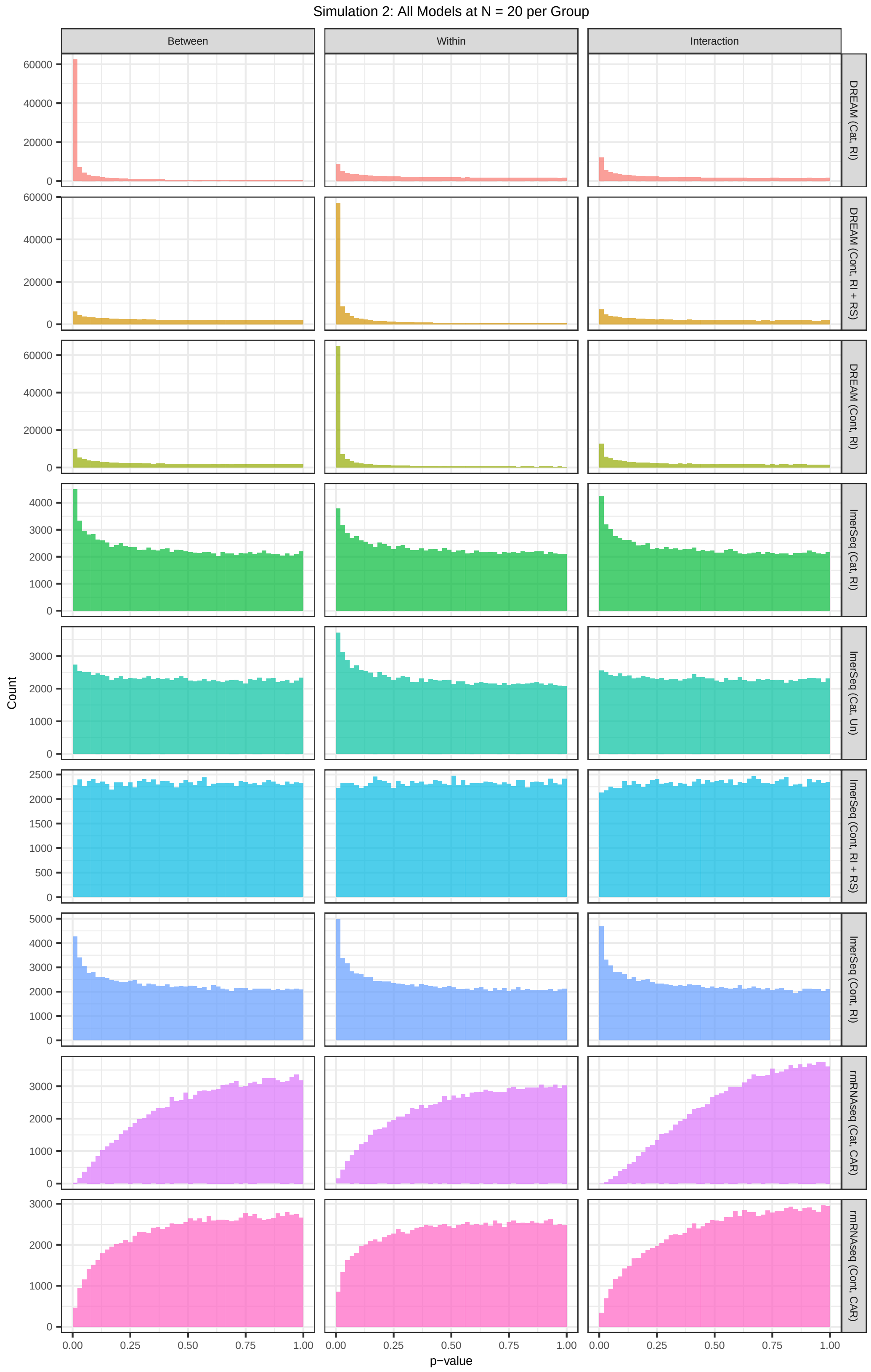

Supplement: Supplementary file 1 — Additional file 1: Supplementary figures S1–S12. [file 12859_2022_5019_MOESM1_ESM.pdf]
